# Synthesis, physical properties, and root canal sealing of experimental MTA- and salicylate-based root canal sealers

**DOI:** 10.1371/journal.pone.0329476

**Published:** 2025-07-31

**Authors:** Rafael Pino Vitti, Kusai Baroudi, Tarun Walia, Raghavandra M. Shetty, Flávia Goulart da Rosa Cardoso, Flávia de Moura Pereira, Evandro Piva, Cesar Henrique Zanchi, Gabriel Flores Abuna, Carolina Oliveira de Lima, Emmanuel João Nogueira Leal Silva, Flávio Henrique Baggio Aguiar, Mário Alexandre Coelho Sinhoreti

**Affiliations:** 1 School of Dentistry, University of Taubaté, Taubaté, São Paulo, Brazil; 2 Department of Restorative Dentistry, Piracicaba Dental School, University of Campinas, Piracicaba, São Paulo, Brazil; 3 Department of Implantology, School of Dentistry, University Santo Amaro, São Paulo, São Paulo, Brazil; 4 Department of Clinical Sciences, College of Dentistry, Ajman University, Ajman, United Arab Emirates; 5 Centre of Medical and Bio-allied Health Sciences Research, Ajman, United Arab Emirates; 6 Department of Pediatric and Preventive Dentistry, Adjunct Faculty, Sharad Pawar College and Hospital, Datta Meghe Institute of Higher Education and Research (Declared as Deemed-to-be University), Wadha, Maharashtra, India; 7 Department of Operative Dentistry, School of Dentistry, Federal University of Pelotas, Pelotas, Rio Grande do Sul, Brazil; 8 Division of Biomedical Materials, School of Dental Medicine, East Carolina University, Greenville, North Carolina, United States of America; 9 Department of Endodontics, School of Dentistry, Grande Rio University, Duque de Caxias, Rio de Janeiro, Brazil; University of Puthisastra, CAMBODIA

## Abstract

**Objectives:**

To develop and evaluate the physical properties and sealing ability within the root canal of three experimental sealers based on MTA and a salicylate resin.

**Materials and methods:**

The experimental sealers were composed of two pastes. The base paste was prepared using 1,3-butyleneglycol disalicylate and bismuth oxide. Three different catalytic pastes were formulated, creating three groups: [MTA] n,n,dihydroxyethyl-p-toluidine (DPT), titanium dioxide (TiO_2_), and mineral trioxide aggregate (MTA); [MTA-HA] DPT + TiO_2_ + MTA + hydroxyapatite (HA); and [MTA-DCPD] DPT + TiO_2_ + MTA + dibasic calcium phosphate dihydrate (DCPD). MTA Fillapex (Angelus) was used as the commercial reference (control). The sealers were manipulated at a 1:1 ratio (base paste:catalyst). Tests for working time and setting time, flow, and film thickness were conducted following ISO 6876:2012 standards. Single-rooted human teeth root canals were utilized for evaluating root canal filling using micro-computed tomography, push-out bond strength testing, and sealer penetration into dentinal tubules using confocal microscopy. Failure patterns in the push-out test were classified as adhesive, cohesive, or mixed. Sealer micromorphology was analyzed via scanning electron microscopy. Data were analyzed statistically (α = 0.05).

**Results:**

MTA Fillapex showed the longest working and setting times, highest flow, the lowest film thickness, and better penetration into dentinal tubules. There was no difference in void among evaluated sealers. Overall, MTA-DCPD sealer showed the lowest bond strength values for cervical and apical thirds. Micromorphological analysis revealed similar crystallographic properties among all sealers.

**Conclusions:**

The sealers tested showed reduced working and setting times, with flow and film thickness according to ISO 6876:2012. Their void volume and bond strength were similar to MTA Fillapex, except for MTA-DCPD. Clinical Relevance: The experimental root canal sealers demonstrated suitable physical properties and good adaptation within the root canal.

## Introduction

The success of endodontic treatment relies on various factors aimed at curing and preventing contamination of the tooth and periapical tissues [1,2]. Once mechanical and chemical cleaning of the root canals is completed, a properly fitted obturation to these canal walls ensures a secure seal, preventing the ingress of microorganisms or tissue fluids [3–5]. Gutta-percha in combination with root canal sealer plays a critical role in establishing the adequate adhesion of the obturation to dentin walls [5].

The quest for biocompatible and bioactive materials in root canal obturation holds promise due to their capacity to induce healing and bone regeneration [6]. Over time, root canal sealers with ion release have been proposed to enhance apical sealing by promoting mineral deposition on the canal walls [6–14]. Calcium-containing root canal sealers constitute a significant category in this context, showcasing good apical sealing capacity and chemical-mechanical interaction with root dentin [6,13,14]. These sealers consist of calcium silicate, calcium aluminate, and other calcium sources [15].

MTA (mineral trioxide aggregate) is a widely used material in dentistry known for its good marginal adaptation, anti-inflammatory properties, and its capability to induce mineralized tissue formation [16]. Despite its advantages, MTA has limitations such as dental discoloration, extended setting time, high cost, and increased cytotoxicity post-manipulation. Hence, several studies have explored the addition of other calcium sources to root canal sealers to enhance their physicochemical and biological properties [11,17–19]. Among these, stoichiometric hydroxyapatite (HA) and dibasic calcium phosphate dihydrate (DCPD) have received considerable attention due to their favorable characteristics, including biocompatibility, osteoconductivity, and satisfactory cellular responses [7–11].

HA exhibits excellent chemical similarity to the mineral phase of human hard tissues, contributing to long-term stability and promoting cell adhesion and proliferation [7,8,10]. Conversely, DCPD is more soluble under physiological conditions and can convert into hydroxyapatite in situ, providing an early burst of calcium and phosphate ions that may stimulate mineralization [9,11,18]. The complementary features of HA and DCPD, such as structural persistence and rapid ionic release, would potentially demonstrate synergistic effects when combined with the salicylate-based formulation tested in this study [11,19,20]. Their inclusion could improve both the initial bioactivity and long-term biological performance of the experimental root canal sealers, making this combination particularly worthy of investigation.

Root canal sealers based on salicylate resins have also been studied and utilized to enhance handling and physical properties. Additionally, these resins demonstrate satisfactory performance owing to their anti-inflammatory properties [21,22]. The combination of different calcium sources with salicylate resin in root canal sealers can enhance their physicochemical properties. Moreover, a higher amount of MTA in these endodontic sealers compositions increases calcium release and pH [11]. New studies assessing the interaction of these calcium sources with salicylate resin could contribute to a better understanding of clinical performance while seeking to improve the physicochemical and biological properties of these materials.

Therefore, the aim of this study was to assess the physical properties and sealing ability of experimental root canal sealers based on MTA and butylene glycol disalicylate. The hypothesis tested was that the experimental root canal sealers compared to the commercial reference (control) would demonstrate superior outcomes in the evaluated properties.

## Materials and methods

### Experimental design

**Fig 1:**
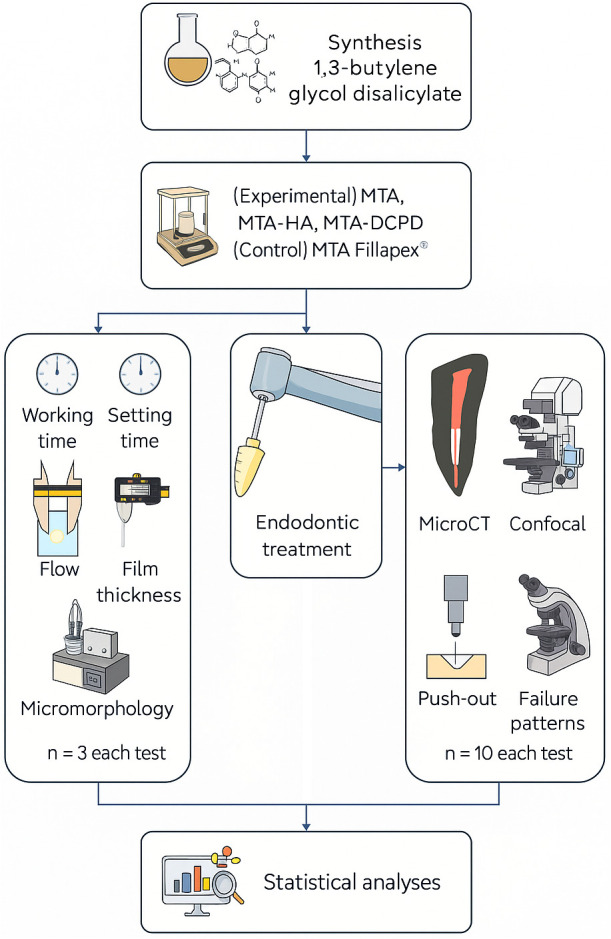
Flowchart of the experimental design.

All procedures performed were in accordance with the ethical standards of the institutional ethics committee approval (CAAE 82540618.9.1001.5501). In this in vitro study, the factors investigated included (i) the type of root canal sealer at 4 levels (three experimental and one commercial sealers) for all tests, and (ii) root canal thirds at 3 levels (cervical, middle, and apical) for the push-out test. The response variables comprised quantitative analysis of working time (n = 3) and setting time (n = 3), flow (n = 3), and film thickness (n = 3); qualitative and quantitative assessment of the percentage volume of void spaces using micro-computed tomography (n = 10) and push-out bond strength (n = 10); as well as qualitative analysis of sealer penetration into dentinal tubules through confocal microscopy (n = 10) and sealer micromorphology using scanning electron microscopy (n = 3) (Fig 1).

### Synthesis of salicylate resin

The salicylate resin (1,3-butyleneglycol disalicylate) was synthesized via transesterification reaction of methyl salicylate (Synth Laboratory, São Paulo, SP, Brazil) with two different alcohols, at a molar ratio of 1:3. Titanium isopropoxide (Sigma-Aldrich, St. Louis, MO, USA) served as the catalyst agent. The reaction was maintained at 200°C for 2 hours. The resulting product underwent purification through vacuum distillation and was characterized via nuclear magnetic resonance (NMR) spectroscopy and Fourier-transform infrared spectroscopy (FTIR), which revealed a distinct peak of hydroxyl groups within the spectral range of 3300 cm^-1^.

### Formulation and handling of root canal sealers

The experimental root canal sealers consisted of two pastes. The base paste comprised 1,3-butyleneglycol disalicylate and bismuth oxide (Vetec, Duque de Caxias, RJ, Brazil). Three catalyst pastes were formulated using different types of calcium phosphate (white MTA, hydroxyapatite, and DCPD): [MTA] n,n-dihydroxyethyl-p-toluidine (DPT; Sigma-Aldrich), titanium dioxide (TiO_2_; Sigma-Aldrich), and MTA (Angelus, Londrina, PR, Brazil); [MTA-HA] DPT + TiO_2_ + MTA + hydroxyapatite (Sigma-Aldrich); [MTA- DCPD] DPT + TiO_2_ + MTA + DCPD (Vetec). The components were measured and weighed on a precision analytical balance. MTA Fillapex (Angelus) served as the commercial reference (control) (Table 1).

**Table 1:** Composition of root canal sealers tested.

Groups	Composition (% weight)
MTA	Base paste: 1,3-butyleneglycol disalicylate (60%) and bismuth oxide (40%). Catalyst paste: MTA (60%), DPT (39%) e TiO_2_ (1%).
MTA-HA	Base paste: 1,3-butyleneglycol disalicylate (60%) and bismuth oxide (40%). Catalyst paste: MTA (40%), DPT (39%), hydroxyapatite (20%) e TiO_2_ (1%).
MTA- DCPD	Base paste: 1,3-butyleneglycol disalicylate (60%) and bismuth oxide (40%). Catalyst paste: MTA (40%), DPT (39%), DCPD (20%) e TiO_2_ (1%).
MTA Fillapex	Base paste: methyl salicylate butylene glycol colophony, bismuth trioxide, fumed silicon dioxide. Catalyst paste: fumed silicon dioxide, titanium dioxide, mineral trioxide aggregate, pentaerythritol, rosinate, p-toluenesolfonamide.

DPT: n,n-dihydroxyethyl-p-toluidine; TiO_2_: titanium dioxide; DCPD: dibasic calcium phosphate dihydrate.

The endodontic sealers were proportioned using equal volumes of the two pastes (base and catalyst) on a glass slab. Manipulation was performed using a metal spatula nº 24 (SS-White Duflex, Rio de Janeiro, RJ, Brazil) for 30 seconds, ensuring thorough homogenization of the two pastes.

### Flow and working time

The flow and working time tests were conducted following the International Organization for Standardization (ISO) 6876:2012 (revised and confirmed in 2017). Following the aforementioned manual manipulation of the tested root canal sealers, 0.05 mL of each sealer was placed at the center of a second glass slab (40 x 40 mm, 5 mm thick, and 20 g). At 180 seconds after the start of manipulation, a third glass slab with the same dimensions and mass was centrally placed atop the sealer, followed by a metallic device weighing 100 g, totaling a mass of 120 g on the sealer. Ten minutes later, the weight was removed, and the maximum and minimum diameters of the compressed disc formed by the sealer were measured using a digital caliper with a precision of 0.01 mm (model 100.170, Digimess, São Paulo, SP, Brazil). The test was repeated if the two diameters showed a difference greater than 1 mm (n = 3). The mean values and standard deviation were calculated and recorded (mm) to obtain the flow values.

New samples were manipulated under the same conditions to evaluate working time (n = 3). Working time was recorded when the sealer reached 90% of the diameter measured in the flow test.

### Setting time

The setting time test was realized following the ISO 6876:2012 specification (revised and confirmed in 2017). Gypsum molds (10 mm diameter, 1 mm thickness) were pre-stored in an incubator at 37°C and 95% relative humidity for 24 hours. The root canal sealers (n = 3) were proportioned and manipulated as previously described. Subsequently, these materials were inserted into the gypsum molds freshly removed from the incubator. A glass slide (1 mm thick) was placed over the mold/sealer assembly for surface leveling.

Following this, an indenter with a 100 g load and a flat-end 2 mm diameter was positioned perpendicular to the specimens and carefully lowered vertically onto the sealer surface. The process was repeated until no indentations were visible on the specimen surface (n = 3). The indenter tip was cleaned before each reading. The time between the start of manipulation and when no visible indentations could be seen on the sealer was recorded as the setting time.

### Film thickness

For the film thickness measurement (n = 3), acrylic plates (50 mm width x 50 mm length x 5 mm thickness) were stacked on top of each other, confirming a total thickness of 10 mm using a digital caliper accurate to 0.001 mm (model 5–30 mm series 345, Mitutoyo, Tokyo, Japan). After manipulating the root canal sealers, they were placed on an acrylic plate, with a second acrylic plate positioned on top of the sealer. Subsequently, a compressive load of 150N (MBio I 500, BioPDI) was applied to this plate-sealer-plate assembly. The thickness of this assembly was then measured again using the caliper with an accuracy of 0.001 mm (Mitutoyo), and the difference between the initial and final measurements was recorded as the film thickness (µm). This test was also conducted in accordance with the ISO 6876:2012 specification (revised and confirmed in 2017).

### Endodontic preparations

A pilot study was conducted to calibrate the operators (FGRC and FMP) and to determine the minimum number of samples per group (sample calculation) for tests not included in the ISO 6876:2012 specification (void and push-out bond strength tests). The sample size was determined based on a pilot study and previous literature, using G*Power software. A power analysis (α = 0.05, power = 80%) was conducted to ensure adequate statistical sensitivity for detecting significant differences between groups.

Extracted human single-rooted canines and premolars for therapeutic reasons were selected in the period between 10 April 2018 and 10 June 2018. Written consent for the use of these teeth was obtained. Initially, standardization of the teeth occurred through macroscopic inspection using a stereomicroscope with 20x magnification (Leica Microsystems, Wetzlar, Germany) to verify intact roots and complete apices. Teeth with carious lesions, cracks, and/or any other lesions in the root were discarded. Teeth with a minimum of 10 mm of root length were included in this study. The teeth were also radiographed to visualize canal anatomy in mesiodistal and buccolingual directions; those showing calcifications, incomplete root apex, or prior endodontic treatment were excluded. The selected teeth were stored in distilled water at 4°C for use within 6 months after Ethics Committee approval from the university of Taubate, Brazil (CAAE 82540618.9.1001.5501). Subsequently, the teeth were sectioned 1 mm above the cementoenamel junction to obtain a flat and deep dentin surface using a low-speed diamond disc under water-cooling (Isomet 1000, Buehler, Lake Bluff, IL, USA).

Root canal irrigation was performed using 2.5% sodium hypochlorite solution, followed by its aspiration and re-insertion. Canal exploration was then performed with Kerr file #15 (Dentsply-Maillefer, Ballaigues, Switzerland) until reaching the apical foramen. The working length was determined for each sample by subtracting 1 mm from the total length traveled by the file during exploration. The canals were further prepared using rotary instruments Mtwo NiTi (VDW; VDW GmbH, Munich, Germany) up to size 40.04 and irrigated with 2.5% sodium hypochlorite solution after each instrument change.

All apices of the samples were sealed with utility wax to allow the flow and reflux of irrigating solutions. Root canal preparation was finalized through instrumentation at the working length. The samples underwent syringe irrigation using Navi Tips (Ultradent Products, South Jordan, UT, USA) positioned 2 mm from the working length. Final irrigation consisted of 5 mL of 2.5% sodium hypochlorite, followed by 5 mL of 17% EDTA (pH 7.7; Odahcam, Dentsply, Petrópolis, RJ, Brazil), and concluded with 5 mL of 2.5% sodium hypochlorite. After the final irrigation, the canals were dried using White Mac (Ultradent) and Capillary Tips (Ultradent) and dried with absorbent paper points corresponding to instrument size 40.04.

Obturation was performed using the single cone technique with 40.04 gutta-percha cones (Mtwo, VDW) previously calibrated on a millimeter ruler. Subsequently, the root canal sealers were manipulated as previously described and spread over the gutta-percha cone surface for insertion into the root canals. Excess gutta-percha was removed at the canal orifice using heated pluggers and cold vertical compression. The quality of obturation was assessed through radiographic imaging. The roots were kept in an incubator at 37°C and 100% humidity for 7 days.

### Micro-computed tomography

The micro-computed tomography procedure was conducted and refined in accordance with previously published protocols [23–26]. Computed tomography imaging was conducted on the obturated teeth (n = 10) using a microtomograph (SkyScan 1173, Bruker, Kontich, Belgium) with exposure parameters set at 70 kV and 114uA. A 1 mm thick aluminum filter was applied, exposure time set at 320 milliseconds, a rotation step of 0.5, and a full 360-degree rotation around the vertical axis. The isotropic resolution was 17 µm. Images were reconstructed using the NRecon software (v1.6.1.0; Bruker, Kontich, Belgium) with specific reconstruction parameters: 1 ring artifact correction, 35% beam hardening correction, and smoothing of 2 for all images.

ImageJ software was utilized for analyzing the volume of obturation material and the presence of voids. The binarization process was employed, involving processing grayscale levels to obtain a black-and-white image exclusively. The range of grayscale required to recognize the obturation material and voids was determined from a density histogram using the global thresholding method. Subsequent arithmetic and logical operations were applied to create separate binary images of the root canal and obturation material.

The voids volume was calculated using the formula:


% voids = void volume x 100/obturation material volume\]

### Push-out bond strength

The samples were fixed onto acrylic plates using sticky wax (Kota, São Paulo, SP, Brazil). This assembly was sectioned using a metallographic cutter (Isomet 1000, Buehler) with a diamond disc (0.03 mm thickness) at low speed (350 rpm) and water cooling. The cuts were made from the root apex towards the cervical portion, and the samples were cut to an approximate thickness of 1 mm in each of the thirds (cervical, middle, and apical). The cervical part of each section was marked with a pen, and the thickness was verified using a digital caliper with an accuracy of 0.001 mm (Mitutoyo). Each root was sectioned into six slices: two apical, two middle, and two cervical. The first slice from each root third was used for the push-out test, while the second slice was used for dentinal penetration (confocal).

Subsequently, the specimens were stored for 3 hours at 37ºC. For the push-out test (n = 10), the first slice was placed into a universal testing machine (Instron 1144, Norwood, USA) at a speed of 0.5 mm/min and a load cell of 500N until displacement of the filling occurred. The tip used touched only the filling mass (sealer and gutta-percha) in the evaluated thirds. The specimen was mounted in the universal testing machine apico-coronally, meaning the smaller diameter was facing upwards and the larger one downwards (the cut part marked with the pen) to prevent any interference caused by the anatomical narrowing of the root canal during the test. The force values required for the displacement of the filling in each specimen (thickness) were obtained in Newtons (N) and converted to MegaPascals (MPa) by dividing the force value by the bonding area of the filling material (mm^2^). To calculate the bonding area, the formula used was:


$$RU=\pi \;(R1+R2)\;{\left[{\left(R1-R2\right)}^{2}\right]}^{1/2}+h^{2}\mathrm{\backslash ]}$$

The π is 3.14, where R1 is the radius of the root opening at the apical face of the root, R2 is the radius of the root opening at the cervical face of the root, and h is the slice thickness.

Each third was examined using a stereomicroscope at 20x magnification (Leica Microsystems) to determine the failure pattern frequency: adhesive (no visible sealer on dentin walls), cohesive (complete dentin walls covered with sealer), or mixed (combination of adhesive and cohesive; partial dentin coverage with sealer).

### Sealer penetration into dentinal tubules

The 0.1% by weight of rhodamine B (Sigma Chemicals, St. Louis, MO, USA) was added in the root canal sealers during their manipulation. The root canals (n = 10) were comprehensively assessed, and representative images of micropermeability patterns (sealer penetration) were recorded. Images were captured using a laser scanning confocal microscope equipped with a 63x oil immersion lens (NA 1.4) and an illumination system comprising an argon/helium laser (488 nm) and a helium-neon laser (633 nm) with absorption and emission wavelengths suitable for rhodamine B and fluorescein. Fluorescence images were acquired from optical sections of 20μm, located 1 mm below the surface (z-axis).

### Sealer micromorphology

All root canal sealers tested were manipulated and inserted into silicone molds with 8 mm internal diameter and 1.6 mm height (n = 3) on a glass plate for microstructure evaluation using scanning electron microscopy (SEM; JSM 5600LV, JEOL, Tokyo, Japan). A polyester strip and a glass slide were placed over the sealer to flatten the surfaces. The samples were kept in an oven at 37ºC for 24 hours. Subsequently, the samples were removed from the silicone molds and placed in a desiccator for complete moisture removal. The specimens were then positioned on a conductive carbon adhesive tape attached to a metal stub for gold sputter coating (Bal-Tec SCD-050 Sputter Coater, Liechtenstein) prior to SEM analysis.

### Statistical analyses

The statistical analyses included preliminary tests to assess the normality of the sample distribution. Upon confirming normality (ρ > 0.05 in the Shapiro-Wilk test) and homoscedasticity (ρ > 0.05 in the Levene test), parametric statistical tests were employed, except for the push-out bond strength data (ρ = 0.020 in the Shapiro-Wilk test). One-way analysis of variance (ANOVA) with Tukey’s post-hoc test (α = 0.05) were used for parametric data. The push-out bond strength data were evaluated using the Kruskal-Wallis and Student-Newman-Keuls test (α = 0.05). Dunnett’s test was employed to compare all experimental groups against the single control group while maintaining strict control of the family-wise error rate. Statistical analysis was conducted using SPSS 22.0 (Statistical Package for the Social Sciences).

## Results

Table 2 shows that the control group exhibited statistically the highest values in working and setting times, as well as in flow (ρ < 0.05). However, regarding film thickness, all experimental root canal sealers showed higher values without statistical differences between them (ρ > 0.05).

**Table 2:** Means (±SD) of working and setting times (min), flow (mm), and film thickness (µm).

Sealer	Working time	Setting time	Flow	Film thickness
MTA	16.2 (0.8) a*	191.7 (14.4) a*	23.87 (0.88) a*	48 (1.48) a*
MTA-HA	15.7 (0.3) a*	183.3 (14.4) a*	22.05 (0.60) a*	45 (1.07) a*
MTA- DCPD	15.3 (0.6) a*	183.3 (38.1) a*	22.37 (0.35) a*	46 (2.01) a*
MTA Fillapex	30.2 (0.8)	258.3 (28.8)	28.35 (0.83)	25 (3.52)

Within each test (column), different letters indicate statistically significant differences between sealers (ρ<0.05). Asterisks (*) denote significant differences between experimental sealers and the control group (MTA Fillapex; ρ<0.05).

**Fig 2:**
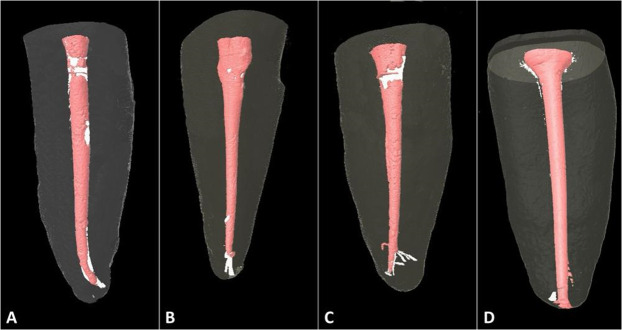
Three-dimensional computed tomography images displaying the filling of root canals by the root canal sealers. (A) MTA; (B) MTA-HA; (C) MTA-DCPD; (D) MTA Fillapex.

In the micro-computed tomography analysis, assessing the entire extension of the root canal, despite minor qualitative differences in root canal obturation (Fig 2), the evaluated experimental groups did not exhibit differences among themselves or with the control group concerning the volume of void spaces (ρ > 0.05) (Table 3).

**Table 3:** Means (±SD) of voids volume in root canals.

Sealer	Voids (%)
MTA	7.37 (9.69)
MTA-HA	8.09 (4.63)
MTA-DCPD	8.47 (8.09)
MTA Fillapex	5.27 (7.54)

For the push-out bond strength test, the Kruskal-Wallis test revealed interaction between the factors of experimental root canal sealers and root thirds (ρ = 0.009), with significant differences observed only for the material factor (ρ < 0.001). Table 4 illustrates that all experimental root canal sealers exhibited satisfactory push-out bond strength values, as their means did not significantly differ from the control group (ρ > 0.05), except for the MTA-DCPD group in the cervical third (ρ = 0.017). No differences in bond strength were observed among the root thirds for all groups (ρ > 0.05).

**Table 4:** Means (±SD) of bond strength (MPa) of root canal sealers and root thirds.

Sealer	Cervical	Middle	Apical	Pool mean
MTA	2.12 (1.20) ab	2.61 (1.60) a	2.59 (1.49) ab	2.44 (1.54)
MTA-HA	4.82 (3.39) a	2.94 (1.14) a	5.92 (4.19) a	4.56 (3.88)
MTA-DCPD	1.24 (0.44) b*	1.91 (0.96) a	2.46 (1.45) b	1.87 (1.20)
MTA Fillapex	2.92 (2.29)	3.07 (2.45)	3.62 (2.75)	3.20 (2.65)

Different letters indicate statistical differences between the sealers (columns) (ρ<0.05). Asterisk (*) indicates statistical differences between the experimental sealers and the control group (column) (ρ<0.05). Control group: MTA Fillapex.

Cohesive failure was predominant for all tested root canal sealers, with MTA Fillapex presenting the highest percentage of cohesive failures (Table 5).

**Table 5:** Distribution of failure patterns of the root canal sealers.

Sealers	Adhesive	Cohesive	Mixed
MTA	13%	70%	17%
MTA-HA	6%	78%	16%
MTA-DCPD	11%	73%	16%
MTA Fillapex	3%	89%	8%

**Fig 3:**
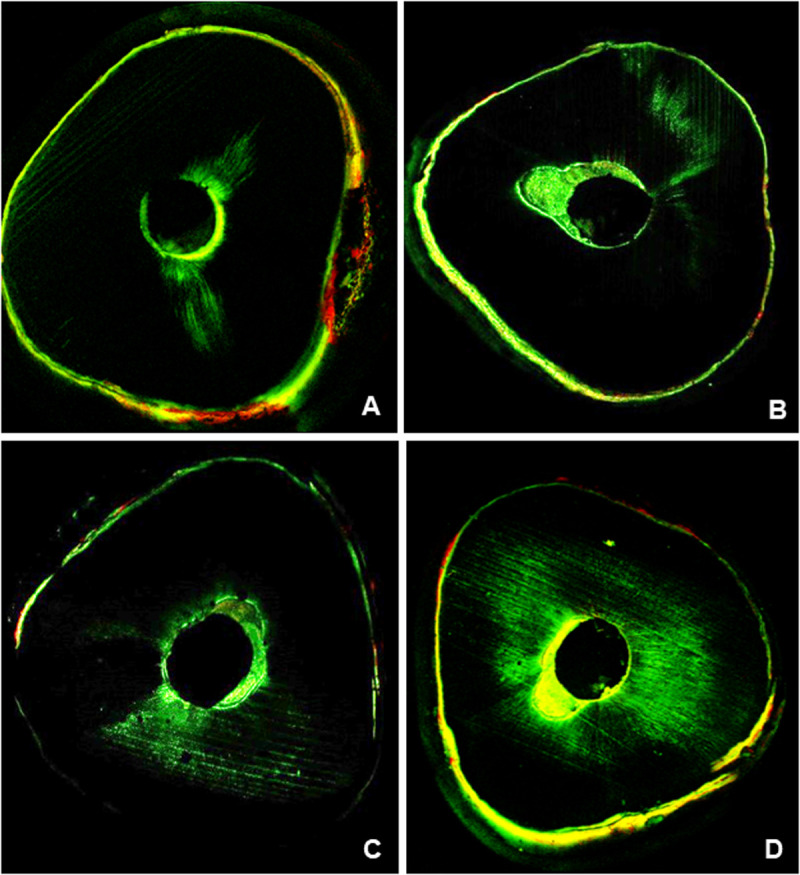
Laser confocal microscopy images showing the penetration of cements into dentinal tubules. (A) MTA; (B) MTA-HA; (C) MTA-DCPD; (D) MTA Fillapex.

Fig 3 comprises representative images obtained by laser confocal microscopy of the cervical section of the root thirds of all groups. The control group (MTA Fillapex) exhibited the most extensive cement penetration into root dentin qualitatively.

**Fig 4:**
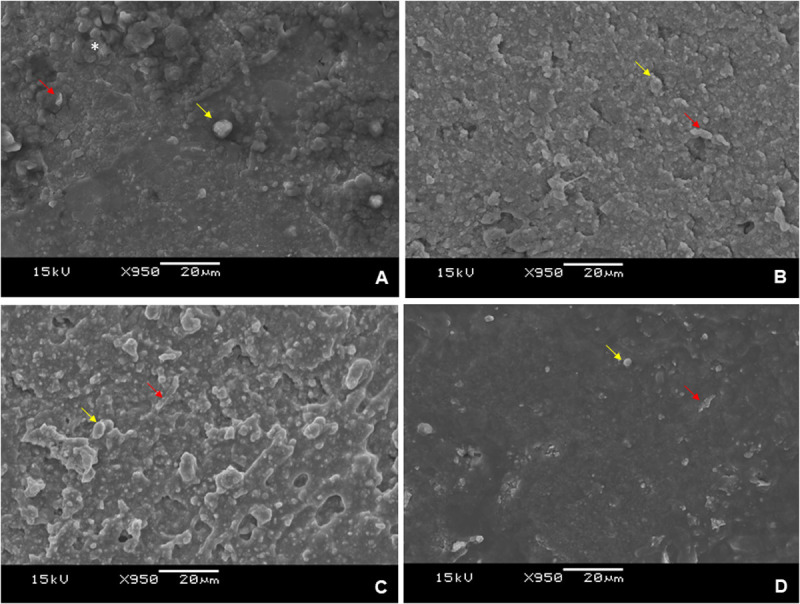
Scanning electron microscopy (SEM) images depicting the micromorphology of the sealers. (A) MTA; (B) MTA-HA; (C) MTA-DCPD; (D) MTA Fillapex. Asterisk: particle cluster. Yellow arrow: cylindrical particle. Red arrow: point particle.

The micromorphology analyses of all root canal sealer samples assessed in this study are depicted in Fig 4. The dominance of amorphous particles is evident for all groups, with the presence of cylindrical particles (yellow arrow) and point crystals (red arrow). Notably in MTA (Fig 4A), there are clusters of particles with varying dimensions (asterisk), whereas in MTA Fillapex (Fig 4D), particles appear more dispersed. The MTA-HA (Fig 4B) and MTA-DCPD (Fig 3C) samples also reveal the formation of cubic-shaped particles, which are larger, more homogeneous, and more abundant compared to MTA Fillapex (Fig 4D).

## Discussion

The hypothesis was rejected since the control group exhibited statistically the highest values in working and setting times, as well as in flow (Table 2). However, regarding film thickness, all experimental root canal sealers showed higher values without statistical differences between them (Table 2).

Both working and setting times are dependent on the chemical composition of root canal sealers, particle size, manipulation temperature, and relative humidity of the environment [11]. All evaluated root canal sealers contain MTA (Table 1) and set through two main chemical reactions: the progressive hydration of orthosilicate ions (SiO_4_^4-^) and the chemical reaction between MTA and salicylate resin [11,20]. During manipulation, these two components come into contact, and calcium reacts with salicylate to create an ionic polymer. Hydration occurs as the tricalcium silicate particles in MTA react with water, forming a hydrated solution of amorphous calcium silicate, taking set, and forming a solid and uniform network [18]. Thus, the differences found in the working and setting times of the evaluated materials can be attributed to the chemical composition of each sealer. The manipulated MTA Fillapex contains approximately 13% MTA in its composition [27]. Thus, a possible explanation for the experimental root canal sealers showing the lowest working and setting times values lies in the MTA-salicylate resin ratio. The higher quantity of MTA present in the experimental materials (40–60% in the catalyst paste; Table 1), around 20–30% of the manipulated sealer mass, accelerates the MTA-salicylate chemical reaction, resulting in shorter working and setting times (Table 2).

The same MTA-salicylate resin ratio, which grants MTA Fillapex longer setting and working times, seems to account for its higher flow in comparison to the experimental sealers (Table 1). A larger amount of salicylate resin makes the material more fluid and consequently increases its flowability. The particle size of MTA also influences viscosity and, consequently, flow. Smaller particles lead to a larger contact area during the reaction with salicylate resin, increasing viscosity and reducing flow [28,29]. MTA Fillapex presents MTA particles with an average size of 12 µm, while the MTA used in the experimental root canal sealers had an average size of 5 µm (manufacturer’s data).

It’s important to highlight that all tested root canal sealers comply with ISO 6876:2012 [30], which recommends that endodontic sealers should have a flow greater than 20 mm. A root canal sealer should demonstrate moderate flow, as excessive flow increases the likelihood of material extrusion into the periapical region. Conversely, low flow reduces sealer penetration into irregularities of the main canal and accessory canals, compromising the material’s sealing ability [31]. All tested sealers exhibit pseudoplastic behavior, where their viscosity reduces while flow increases. This occurs due to the shear forces acting on the sealer during its application [31,32].

The MTA-resin salicylate ratio in the experimental root canal sealers is sufficient for these materials to exhibit a film thickness within the limit required by ISO 6876:2012 (≤ 50 µm) [30]. This parameter is crucial for proper distribution of the root canal sealer within the root canals [33], particularly during the insertion of gutta-percha [5,34]. The increased film thickness of the experimental root canal sealers might be associated with the size of the MTA particles, as the particle size in these materials generates a larger surface area and increases viscosity, as mentioned above. Additionally, the amount of radiopacifier (bismuth oxide) used in these materials may influence material hydration, affecting their film thickness [35]. Bismuth oxide has a high molecular weight, and its presence alters the physical properties of sealers, leading to internal flaws and increased thickness [36]. MTA Fillapex contains approximately 10.5% bismuth oxide [37], while the experimental root canal sealers used in this study had 40% in the base paste (Table 1), totaling around 20% of the manipulated sealer.

The filling of root canals and the presence of voids, measured by micro-computed tomography, showed minor qualitative differences in root canal obturation among the groups (Fig 2). Furthermore, the experimental groups did not exhibit differences in void spaces compared to the control group (Table 3). This indicates that all experimental root canal sealers demonstrated satisfactory performance in filling the root canals.

The presence of voids within the root canal post-obturation emerges as a critical factor that may compromise the clinical prognosis of endodontic treatment [23]. This condition significantly hampers the sealing of the root canal, increasing the potential for periradicular tissue fluid movement into the root canals. Moreover, the presence of voids can facilitate the entry of microorganisms into the periradicular tissues, potentially triggering or perpetuating apical periodontitis [24,34]. Analysis using computerized microtomography, encompassing the entire length of the root canal, did not reveal significant differences in voids volume (Table 3). These findings confirm that all experimental root canal sealers displayed satisfactory performance in root canal filling, with values comparable to the control group.

The experimental and commercial root canal sealers evaluated are MTA-based and have a similar chemical composition [11]. An important aspect to consider is that the evaluated teeth were single-rooted, and the obturation technique employed was single-cone with vertical compaction. Previous studies have emphasized that the technique used in obturation can significantly impact the quality of the procedure [38].

The proper obturation achieved by all experimental root canal sealers, due to the low formation of voids, might have positively influenced the push-out bond strength values. However, it’s important to note that the best bond strength result doesn’t always reflect complete filling of the root canal space [25]. This might explain why all evaluated sealers showed similar volumes of voids (Table 3) and, at the same time, different push-out bond strength values (Table 4). Cohesive failures were predominant for all root canal sealers (Table 5). This could be explained by its thinner film thickness compared to the experimental sealers (Table 2). A greater thickness and cohesion of the sealer are linked to better adhesion to the root canal, as a thin layer of root canal sealer with low cohesive strength is more susceptible to contraction and material displacement [39]. These results, in comparison with literature data, suggest that besides the quantity of voids, the push-out bond strength results from a combination of factors, including root canal anatomy, obturation technique, and mainly the physicochemical properties, particle amount, viscosity, and flowability of the root canal sealers [26,40].

Although the MTA-HA group demonstrated significantly higher bond strength values, its performance remained comparable to MTA group in all root thirds, and all experimental sealers exhibited satisfactory results, comparable to the control group (Table 4). The superior performance of MTA-HA may be attributed to the synergistic interaction between MTA and hydroxyapatite, which enhances interfacial adaptation, cohesive strength, and bioactivity [6–11,18,19]. Notably, SEM analysis revealed uniformly distributed cubic-shaped particles within the MTA-HA microstructure (Fig 4B) may have contributed to mechanical interlocking and material stability [11,19].

The penetration of root canal sealers into dentinal tubules may exhibit a bactericidal effect by establishing contact with residual bacteria residing within these tubules. Furthermore, the presence of sealers in dentinal tubules provides a mechanical interlocking, enhancing the retention of sealer within the root canal [32]. The penetration of root canal sealer into the root canal is influenced by various factors, including the efficacy of smear layer removal, the presence of moisture, the number and diameter of dentinal tubules, the anatomy of the root canal system, as well as the physicochemical properties of the sealers themselves [41]. Thus, both the volume of void spaces (Table 3 and Fig 2) and the depth of sealer penetration into dentinal tubules (Fig 3) may influence the success of endodontic therapy.

MTA Fillapex exhibited extensive cement penetration into the root dentin (Fig 3). The alkaline nature of this sealer denatures collagen fibers, facilitating its penetration [42]. Additionally, good sealer penetration is related to its high flowability, inherent to the chemical composition and smaller particle size of MTA-based sealers [28,29]. The higher flowability (Table 1) and the higher molar ratio between calcium and phosphorus atoms (Ca/P ratio) in MTA Fillapex (Ca/P ~ 2.5) [43], compared to the tested experimental root canal sealers, may explain the superior results in the control group.

Fig 4 illustrates that all tested root canal sealers contain a significant amount of amorphous particles in cylindrical and point crystal forms. In the MTA group, there are clusters of particles with varying dimensions (Fig 4A). In contrast, these particles appear more dispersed in the MTA Fillapex group (Fig 4D). Additionally, the MTA-HA (Fig 4B) and MTA-DCPD (Fig 4C) groups exhibit cubic-shaped particles. Larger particles tend to have a smaller surface area (contact area) of the sealer, consequently affecting the material’s working and setting times (Table 1) [28,29]. These particles observed in the SEM images are presumed to be isolated or agglomerated particles of MTA, hydroxyapatite, and DCPD. Further analysis involving energy dispersive x-ray spectroscopy (EDS) coupled with SEM is warranted to confirm the composition of these particles.

The SEM images provided insights into the structural morphology of the particles present in the evaluated sealers. The size and geometry of these particles significantly influence the sealer hydration kinetics [15,18]. The hydration process initiates following the dissolution of the powder (MTA, HA, and DCPD) and the subsequent crystallization of particles, characterized by the presence of cubic and point crystals (Fig 4). Despite SEM analysis provided relevant micromorphological insights, additional compositional characterization using EDX or XRD could have enhanced the understanding of the chemical nature and crystalline structure of the sealers. Future studies are encouraged to explore these techniques to complement the current findings.

Although the present study did not directly assess the bioactivity of the experimental sealers, their composition allows for some considerations in this regard. First, the incorporation of MTA, HA, and DCPD in the experimental formulations may favor cellular compatibility and interaction, as similar calcium phosphate-based materials have demonstrated good biocompatibility and the ability to support favorable cellular responses in vitro and in vivo [7,8,10,11]. Second, calcium-releasing materials such as MTA and DCPD have been associated with mild antimicrobial effects, especially against *Enterococcus faecalis*, due to their ability to elevate pH and create an unfavorable environment for bacterial survival [9,11]. Additionally, the release of calcium and phosphate ions from these formulations may enhance the nucleation of calcium phosphate phases and contribute to hydroxyapatite formation, a key indicator of bioactivity [6,9,18]. Finally, although this study did not employ simulated body fluid or assess apatite precursor formation directly, previous investigations using similar materials have reported the formation of apatite-like crystals when immersed in supersaturated phosphate solutions, supporting their potential bioactive behavior [18,19]. These aspects suggest that the experimental sealers tested here may exhibit promising bioactivity, which warrants further investigation in future studies.

The findings of the present study associated with outcomes from other researchers assessing similar root canal sealers [11,22,44], demonstrate that the evaluated experimental root canal sealers exhibit satisfactory physical properties and root canal sealing. However, future investigations are required to assess the esthetic compromise of these materials in anterior teeth, understand the clinical impacts of these experimental sealers on the longevity of endodontic treatment, especially when applied using different obturation techniques in multi-rooted teeth, and in endodontic retreatment.

## Conclusion

Overall, based on the presented results, it can be concluded that the experimental root canal sealers exhibited adequate physical properties and adaptation within the root canal. They (i) demonstrated reduced working and setting times compared to MTA Fillapex, (ii) showed flow and film thickness within the values recommended by ISO 6876:2012, and (iii) exhibited a volume of voids and bond strength similar to MTA Fillapex, except for the MTA-DCPD group in the cervical third.

## Supporting information

S1 File Ethical committee approval report. (PDF)

S2 File Human Research subject checklist. (DOCX)
