# Supplementary material for: Synthesis, physical properties, and root canal sealing of experimental MTA- and salicylate-based root canal sealers
Source: PLoS One. 2025 Jul 31;20(7):e0329476. doi: 10.1371/journal.pone.0329476 (PMC12312910; doi:10.1371/journal.pone.0329476)
Supplement: S1 File — (PDF) [file pone.0329476.s001.pdf]

SUBSTANTIATED OPINION FROM THE ETHICS COMMITTEE

RESEARCH PROJECT DETAILS

Research Title: Development and evaluation of the physical properties of experimental MTA-based endodontic cements and a salicylate resin

Principal Investigator: Rafael Pino Vitti

Thematic Area:

Version: 2

CAAE: 82540618.9.1001.5501

Proposing Institution: University of Taubaté

Primary Sponsor: Self-funded

OPINION DETAILS

Ethics Committee Number: 2.535.649

Project Overview:

The type of salicylate resin used in this study will be 1,3-butyleneglycol disalicylate, synthesized via the transesterification reaction of methyl salicylate with two different alcohols in a 1:3 molar ratio. Titanium isopropoxide will be used as a catalyst for this chemical reaction. The experimental cements will consist of a base paste and a catalyst paste, which will be mixed in equal portions to form a homogeneous mass. The base paste will be composed of bismuth oxide and 1,3-butyleneglycol disalicylate. Three different catalyst pastes will be formulated, varying in the type of calcium phosphate used: (1) n-ethyl o,p-toluene sulfonamide (RP), titanium dioxide (TiO_2), and mineral trioxide aggregate (MTA); (2) RP + TiO_2 + MTA + hydroxyapatite; (3) RP + TiO_2 + MTA + dicalcium phosphate dihydrate. MTA Fillapex (Angelus, Londrina, Brazil) will be prepared according to the manufacturer's instructions (commercial reference). All materials will be weighed and proportioned using an analytical balance with an accuracy of 0.0001 g and manipulated under controlled environmental conditions (23°C ± 2°C and 50% ± 10% relative humidity). A total of 72 recently extracted, caries-free, single-rooted maxillary teeth will be obtained from the Human Teeth Bank of the Department of Dentistry at the University of Taubaté (BDH-D/Unitau) and approved by the Research Ethics Committee of the School of Dentistry at the University of Taubaté. The teeth will be standardized through macroscopic inspection to ensure intact roots, complete apices, and curvatures of less than 5°. Radiographs will also be taken to assess the canal anatomy, and teeth with calcifications, incomplete root apices, or previous endodontic treatment will be excluded. The teeth will be cleaned using curettes to remove hard and soft tissue deposits on the root surface, then stored in a 0.1% thymol suspension (by weight) at 4°C and used within four months after extraction. Prior to use, the teeth will be removed from storage, rinsed under running water to eliminate any traces of

thymol, and dried with paper towels. The teeth will be randomly distributed among the experimental groups. All root canals will be filled using the single cone technique with a 50/05 taper (ProTaper Universal, Dentsply-Maillefer, Ballaigues, Switzerland) and each group will be filled with the corresponding experimental cement. After filling, the crowns will be sealed with Coltosol (Coltene, Switzerland), and the samples will be stored in moistened gauze for 72 hours at room temperature to allow the cements to set. The human teeth will be used for the following analyses: micro-computed tomography (12 teeth), bond strength and adaptation (40 teeth), and microstructure evaluation (20 teeth).

Research Objectives:

The specific objectives of this project are: 1) to synthesize 1,3-butyleneglycol disalicylate through a transesterification reaction; 2) to use 1,3-butyleneglycol disalicylate in the synthesis of experimental MTA-based endodontic sealers with different phosphates (HA and DCPD); 3) to evaluate various physical properties of three experimental endodontic sealers for obturation.

Risk and Benefit Assessment:

The potential risks and benefits were assessed in the project, but not in the informed consent form (ICF), as an exemption from applying the ICF was requested from the Ethics Committee, given that the study will use teeth obtained from the University of Taubaté's Human Teeth Bank.

Comments and Considerations on the Research:

The project is well-designed.

Considerations Regarding Mandatory Presentation Terms:

All mandatory presentation terms were included.

Recommendations:

The Ethics Committee of the University of Taubaté recommends submitting the final report upon completion of the research.

Conclusions or Outstanding Issues and List of Inadequacies:

The outstanding issues from the previous report have been addressed.

Final Considerations by the Ethics Committee:

The Ethics Committee of the University of Taubaté, in a meeting held on 03/09/2018, and in accordance with the competencies defined by CNS/MS Resolution 466/12, has considered the research project:

APPROVED.

Opinion Status:

Approved

Requires CONEP Review:

No

TAUBATE, March 09, 2018

Signed for:
José Roberto Cortelli
(Coordinator)

**Endereço:** Rua Visconde do Rio Branco, 210			
**Bairro:** Centro		**CEP:** 12.020-040	
**UF:** SP	**Município:** TAUBATE		
**Telefone:** (12)3635-1233	**Fax:** (12)3635-1233	**E-mail:** cepunitau@unitau.br	
